# Towards environmental sustainability in Russia: evidence from green universities

**DOI:** 10.1016/j.heliyon.2020.e04719

**Published:** 2020-08-24

**Authors:** Ernest Baba Ali, Valery P. Anufriev

**Affiliations:** a Institute of Economics and Management, Ural Federal University Named After the First President of Russia B.N. Yeltsin, Russia

**Keywords:** Environmental analysis, Environmental economics, Environmental management, Environmental pollution, Education, Sustainability, Environment, Universities, GM ranking, Fixed-effects, Random-effects, Russia

## Abstract

Universities all over the world are increasingly recognizing the need for the attainment of environmental sustainability on campuses resulting in the adoption of several environmental sustainable initiatives by university management. This article, therefore, seeks to investigate the impact of campus-base management practices on environmental quality among Russian Universities. The study also explores the strengths and weaknesses against the best practices for campus sustainability as defined by the UI greenMetric world university ranking as well as the awareness of students about pro-environmental behaviours on campuses. Secondary data from the 2015 to 2019 world university ranking was sourced for the impact assessment as well as the strengths and weaknesses. The study also sourced primary data with the help of a structured questionnaire from student respondents to assess their awareness of pro-environmental behaviours. Fixed-effects, and random-effects models were used to assess the management impact on environmental quality. The result from the empirical models indicate that education and research, transportation and waste influence environmental quality on university campuses. The strengths and weaknesses of universities were assessed based on six (6) categories (setting and infrastructure, energy and climate change, waste, water, transport, and education and research). The study also assessed the perception of students on pro-environmental activities. While the results show an increasing trend of awareness among Russian universities (Management) in campus sustainability initiatives, most students appear to be unaware of environmental initiatives undertaken by their universities. The study, therefore, made some recommendations that can help improve campus sustainability levels while at the same time increase student participation.

## 1 Introduction

The concept of sustainability as a means of dealing with the destruction of the environment as well as mitigating the effect of climate change has long existed since the Stockholm Declaration in 1972 [1]. Since then the concept has evolved taking into account other aspects of the environment ranging from educational institutions to industries and from national economies to the global economy. This has led to the adoption of the concept by different sectors to address environmental challenges. For instance, the concept of sustainability in the educational sector was formally adopted by the Talloires Declaration in 1990 [2], attracting the attention of academics leading to the expansion of literature and the eventual establishment of the UI GreenMetric World University ranking.

In recent times, the green economy concept has been perceived by global organizations such as the World Bank and the United Nations Environment Programme (UNEP) as a pathway to sustainability [3]. However, the ability of a country to fully and effectively transition to a green economy is dependent on the ability to decentralize the concept to involve all institutions (both private and public) of an economy. As such to champion this ideology, universities worldwide have focused their attention on institutional reforms such as the restructuring of curriculum and research as well as introducing campus-based sustainability programs into their daily activities [4]. These reforms undertaken by universities worldwide aims at reducing the effect of anthropogenic activities on the environment as well as developing a future workforce that is environmentally conscious because universities are an essential change agent both in the national and global economy who can produce future leaders that are capable of playing critical roles in achieving environmental sustainability.

The demand to incorporate sustainability practices has gained a lot of attention and acceptance in recent years as this is evident in the increasing number of participating institutions in the UI greenMetric World University Ranking. In light of this, the 2019 ranking featured 46 Russian higher education institutions highlighting the importance of campus sustainability in the country.

Previous studies have assessed the role of universities in the attainment of sustainability of societies [2,4,5]. Most of these studies focused on student base assessment, the strengths, and weaknesses of the green metric ranking as well as assessing university management practices in Africa and other developing countries. Others have investigated students' awareness and valuation of campus-based environmental sustainability [6, 7, 8, 9, 10, 11, 12]. Er and Riwathi [13], developed a conceptual framework for assessing campus sustainability. Employing regression analysis, Presekal et al. [14], evaluated electricity consumption and carbon footprint of participating universities of greenMetric ranking. Despite the growing interest in campus-based sustainability, no studies to the best of knowledge of the authors exist in the specific case of Russian universities regarding the impact of management practices on the improvement of environmental quality. This study, therefore, contributes to the extant literature by assessing the impact of campus-based management practices on environmental quality among Russia universities. Also, the study assessed students' perception of campus-based pro-environmental activities. This was to help provide some basic perspective on student efforts that complement management practices on campus environmental sustainability efforts.

## 2 Literature review

Sustainability on campuses as a road map to the attainment of overall environmental sustainability is gaining a lot of global attention due to the increasing threat climate change poses to the global environment and economy as a whole. As a result higher education institutions worldwide have adopted different strategies and approaches for the attainment of campus sustainability. These strategies and approaches range from water to waste management, green building technologies to renewable energies, and educational reforms to research.

### 2.1 Waste, energy, climate change and variability

Waste generation has rapidly increased over the years especially in developing countries where the normal practice is to buy use and dispose off with little focus on recycling. According to the [15], globally, waste generated annually is about 2 billion tonnes with about 33% of that poorly managed. Globally waste generation per person ranges from 0.11kg to 4.54 kg with an average of 0.74 kg. At this rate of waste generation, it is expected that waste will grow up to 3.40 billion tonnes by 2050 [15].

Developing effective and efficient management of waste system requires an in-depth understanding of the life cycle of waste generation. This includes but not limited to how waste is generated and collected to quantifying the amount of waste generated and the rate of waste generation. As observed by [16], methodologies used for waste collection and mapping include but not limited to records on recycling and visual solid waste management. Given that educational institutions have a vital role to play in the attainment of environmental sustainability, they require infrastructure for waste management systems that match up to the standard of small cities [17]. This does not only help to green the environment but also to practically educate students through a participatory approach. Literature found that different institutions have adopted different approaches to manage campus waste. For instance [17], observed that the Asian Institute of Technology in Thailand developed the “Sustainable Living Laboratory” concept under which a solid waste management program was launched with student volunteers managing the program with assistance from staff. In this model, all waste is dumped in one waste collection bin before they are hand sorted into different bins depending on the type (Recyclable and non-recyclable) waste. In contrast [18] noted that Shenyang University in China adopted a waste management system where waste is separately collected by replacing the traditionally unmarked waste bins with newly designed and marked waste bins (recyclable and non-recyclable). The authors observed that by complimenting this effort with environmental lectures, there was a significant improvement in waste collection and management.

The International Panel on Climate change defines climate change as the significant change in climate variables (rainfall and temperature) of a given location over a given period [19]. Climate change has for a very long time been a global issue with many experts linking the cause to human-factors [20]. Hertwich and Peters [21] showed that daily household consumption and production decisions contributed to about 72% of global emissions. According to [2], about 40% of net global energy is consumed by building and construction leading to the release of about two-third of the total greenhouse gases. Studies have found that energy consumption by educational institutions significantly influences the amount of greenhouse gases in the atmosphere. As noted by [22], higher educational institutions in china alone consume about 40% of the country's public sector energy with the energy consumption level of students more than double that of residents. As a result, the Chinese government took drastic measures to promote the establishment of “green campuses” to help universities to meet sustainable environmental demands. This according to [22], was achieved by:

• Educating future professionals and enhancing students' skills and knowledge on sustainable development.
• Improving energy efficiency on campus.
• Shifting to renewable energy sources, food and other materials used on and off campuses
• Increasing the university's social responsibility for environmental protection and resource economization.
• Broadening the visions/knowledge/opportunities for action on all global environmental issues.

This, however, deals with only one aspect of campus sustainability (energy consumption) and therefore requires the incorporation of other aspects to ensure a holistic approach is adopted [23, 24, 25, 26].

### 2.2 Sustainability assessment tools

Incorporating sustainability into the daily activities of higher education institutions has become very important due to the environmental impact of campus-based medium and large scale operations which greatly affect life both in the internal and external environment [27]. The role of higher education institutions in championing sustainable development on campuses since it first featured in the 1990 Talloires Declaration has been very pronounced in recent years. According to [4], prominent features for campus-based sustainable development were highlighted in global agreements and treaties such as the “Agenda 21 in 1992, the Kyoto Declaration in 1993, Global Higher Education for Sustainability Partnership in 2000, the Luneburg Declaration in 2001, the Sapporo declaration in 2002, Graz Declaration in 2005, Abuja Declaration on Sustainable Development in Africa in 2009, the Rio+20 Higher Education Sustainability Initiative, as well as the United Nations Decade for Education for Sustainable Development”. These agreements have become necessary due to the roles higher education institutions play in fostering learning processes on decision making for both the short and long term [28].

In light of this, several tools have been developed to assess sustainability on campuses [29, 30, 31, 32], however, most of these sustainability assessment tools have had their limitations [33]. For example, while some tools consider only a few sustainable indicators [34], other assessment tools are limited to specific situations and hence cannot be applied to other situations [25,35]. However, some world-known assessment such as GREENSHIP developed in 2008 by Green Building Council of Indonesia consisting of 6 indicators (Table 1) and the Sustainability, Training, Assessment and Rating System (STARS) developed in 2006 in the United States have been used on a large scale for global sustainability rakings (Table 1).

**Table 1:** Study variables and their definition.

Variable	Definition
Energy and Climate change	Improvement in campus-based environmental quality
Setting and infrastructure	Development of campus-based green technology
Waste	Development of sustainable waste management systems on campuses
Water	Sustainable use and management of water campus
Transport	Development of environmentally friendly transport systems of campuses
Education and research	Development of education and research strategies towards environmental sustainability

**Figure 1:**
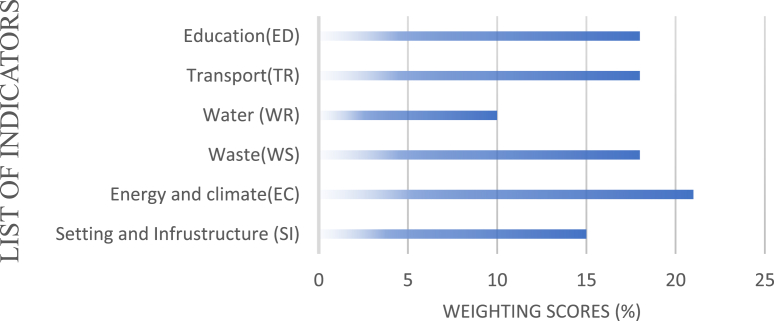
The 2019 UI GreenMetric Ranking and their weighting (UI GreenMetric, [36]).

As noted by [32], these tools also have limitations in that they contain certain indicators that are irrelevant to developed countries hence limiting their applicability in some countries. Also, the complexity in completing some metrics in these tools makes it less user friendly and as such limits participation. These limitations influenced the development of the University of Indonesia World University Ranking for Campus Sustainability (UI GM ranking). Even though the UI GM ranking has undergone some improvements since the initial version was developed in 2010, the ranking still requires some improvements such as incorporating a ranking band that can help differentiate between the levels in sustainability. Nonetheless, the ranking has been wildly accepted as an all-inclusive global ranking system for universities around the world. The ranking consists of 6 broad categories (Figure 1) under which several indicators can be found.

### 2.3 Water, and transport

From Figure 1, Water usage on university campuses is seen as an important indicator in the UI GreenMetric ranking. The aim is to encourage universities to decrease groundwater usage, increase conservation programs, and protect the habitats [36]. Water conservation programs, water recycling programs, water-efficient appliances usage, and treated water usage are among the criteria. Transportation systems play an important role in carbon emission and pollutant levels in universities. Transportation policies to limit the number of motor vehicles on campus include uses of campus buses and bicycles are likely to encourage a healthier environment. For example, a pedestrian policy will encourage students and staff to walk around campus and avoid using private vehicles. This is believed to help decrease emissions levels and improve environmental friendliness.

## 3 Empirical model and data source

### 3.1 Data

In this study, we used panel dataset based on six indicators from the UI greenMetric world university ranking covering 16 Russian universities from 2015 to 2019. Thus, the panel variable is university, and the time variable is in years. The universities covered under the study are RUDN University, Perm University, Minin University, Tomsk P. University, Altai S. University, Don S.T. University, Gorno A.S. University, Saint-P. S.F University, Penza S. University, Voronezh S. University, Polzunov A.S.T University, Russian S.V.P. University, Astrakhan S. University, Tomsk State University, Tver State University, Petrozavodsk State University. These universities were selected for the study because of their consistency in participation in the UI greenMetric ranking from 2015. It is important to note that one major limitation of the dataset is that even though the ranking dates back to 2010, the lack of consistency in participation from participating universities made it impossible to source data from the initial ranking year for most universities.

In light of this, the study evaluated the impact of management practices towards the improvement of campus-based environmental quality among Russian Universities. The UI GreenMetric ranking was considered because of its global acceptance among universities as the best available and user-friendly ranking tool for campus sustainability.

For the purpose of this study, scores assigned to the sustainability indicators based on the GM ranking methodology were used to estimate the effect of the categories on campus-based environmental quality. As indicated by the GM ranking methodology, under each category, there are several indicators on which data is collected and measured. For further details on the calculation and measurement of the categories considered in this study, see [37]. Campus-based environmental quality is a proxy for energy and climate change. It is a measure of the reduction in campus-based CO_2_ emissions. However, based on the GM methodology, emission reduction values are multiplied by a scalar factor to generate ranking scores [37]. Table 1 presents variables and their definitions as used in the study.

In order to assess students awareness and likelihood of participation in pro-environmental behaviours, an online survey using a structured questionnaire was conducted among students across five greenMetric participating universities. The survey was restricted to greenMetric participating universities because of their track record in campus sustainability. A total of 200 questionnaires were sent through mobile contacts, however, only 105 feedbacks were received. As such the sample size for the assessment of students' awareness and likelihood of participation in pro-environmental behaviours was 105. Respondents of the questionnaire included university students across all levels except first year students. We believe first year students are not well placed to be well informed of environmental activities on campuses, hence their exclusion. It is important to note that the institutions associated with this study did not require us to have any ethical approval for this study hence none was received.

### 3.2 Data analysis

Fixed-effects and random-effects regressions are techniques for analyzing panel data [38, 39]. Tests performed are Hausman test and Breusch-Pagan Lagrange multiplier (LM) test for random-effects. Based on the results from the tests, the study used both fixed-effects and random-effects GLS regressions. The outcome variable in the fixed-effects, and random-effects regressions is energy and climate change (see Table 1 for definition) and the predictor variables are setting and infrastructure, Waste, Water, Transport, Education, and research.

#### 3.2.1 Fixed-effects model

The fixed-effects model removes the effect of time-invariant characteristics to assess the net effect of predictors on the outcome variable [40,41]. It also assumes that time-invariant characteristics are unique to a variable and should not be correlated with other variable characteristics [39,42]. Each university is different; hence, the entity's error term and the constant should not correlate with others. If the errors are correlated, the fixed-effects model becomes unsuitable since it may make incorrect inferences; thus, such a relationship may be modeled with the random-effect model. This forms the basis for the Hausman test [40]. Following Torres-Reyna [40], the fixed-effect model is expressed below:

(1) $$Y_{it}=\beta_{0}+\beta_{1}X_{it}+\beta_{2}X_{it}+\alpha_{it}+u_{it}$$

where; $Y_{it}$ denotes the outcome variable (energy and climate change). The subscript *i* denotes entities or panels (the 16 Russian Universities). The subscript *t* denotes time variable (years). $\beta_{0}$ denotes the constant term. $X_{it}$ denotes predictor variables (setting and infrastructure, waste, water, transport, education, and research). β denotes coefficients for predictor variables and they are parameters to be estimated. $\alpha_{it}\;\left(i=1...n\right)$ denotes the unknown intercept for each university (*n* entity-specific intercepts), and $u_{it}$ denotes the error term.

#### 3.2.2 Random-effect model

Unlike fixed-effects, the variation across entities (universities) in the random-effects is assumed to be random and uncorrelated with the predictor variables [40,43]. If variations across entities affect the outcome variable, random-effect model becomes appropriate. The random-effect model assumes that the entity's error term is not correlated with the predictors [40]. Inferences from the random-effect regression can be generalized beyond the sample used for the regression. Following Torres-Reyna [40], the random-effect model is expressed below:

(2) $$Y_{it}=\beta_{0}+\beta_{1}X_{it}+\beta_{2}X_{it}+\alpha_{it}+u_{it}+\varepsilon_{it}$$

where; $u_{it}$ denotes the between-entity error term; and $\varepsilon_{it}$ denotes the within-entity error term. All other variables are as previously defined.

## 4 Results and discussion

### 4.1 Descriptive statistics

The descriptive statistics of the variable are shown in Table 2.

**Table 2:** Descriptive statistics of variables.

Variable name	Count	Mean	Median	Max	Min	Std. dev
Energy and climate change	80	805.9	775	1550	161	291.941
Setting and infrastructure	80	674.525	666.5	1400	165	241.951
Waste	80	829.575	774	1500	198	345.105
Water	80	324.575	300	775	30	157.688
Transportation	80	802.825	858	1650	156	307.620
Education and research	80	682.363	681	1500	43	325.886

**Figure 2:**
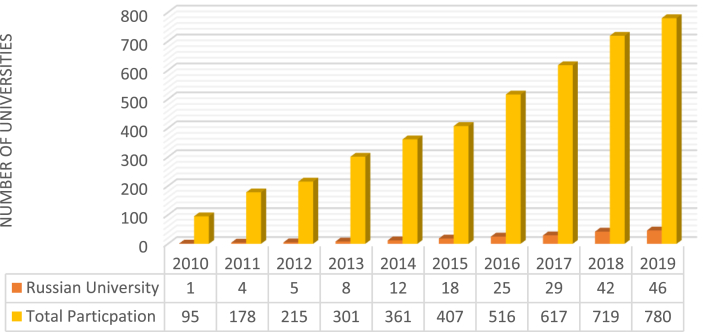
The number of participating Russian universities compared to the overall participation.

Figure 2 shows the progress made by participating universities from Russia over the last five years. It can be observed that the number of participating countries increased over the years implying that campus sustainability (green campus) is gradually being seen as a road map for the attainment of environmental and economic sustainability as a whole.

Test result of the pairwise correlation is shown in Table 3. The maximum correlation among the variables is 0.54 and the minimum is 0.19. Most of the correlation coefficients among most of the variables are less than 0.5 indicating a low correlation among variables. This indicates the avoidance of multicollinearity in the model [44].

**Table 3:** Results of Pairwise correlation test.

	Energy and climate	Setting and Infrastructure	Education and Research	Transportation	Waste management	Water management
Energy and climate	1					
Setting and Infrastructure	0.4508∗ ∗∗	1				
Education and Research	0.5331∗ ∗∗	0.4709∗(0)	1			
Transportation	0.5439∗∗∗	0.4874∗∗∗	0.5276∗∗∗	1		
Waste management	0.4285∗∗∗	0.2256∗ ∗	0.194∗∗	0.488∗∗∗	1	
Water management	0.4084∗∗∗	0.4580∗∗∗	0.299∗∗∗	0.445∗∗∗	0.526∗∗∗	1

Note: ∗,∗∗, and ∗∗∗ denote significance at 10%, 5%, and 1% respectively.

### 4.2 Trends of variables in the study

**Figure 3:**
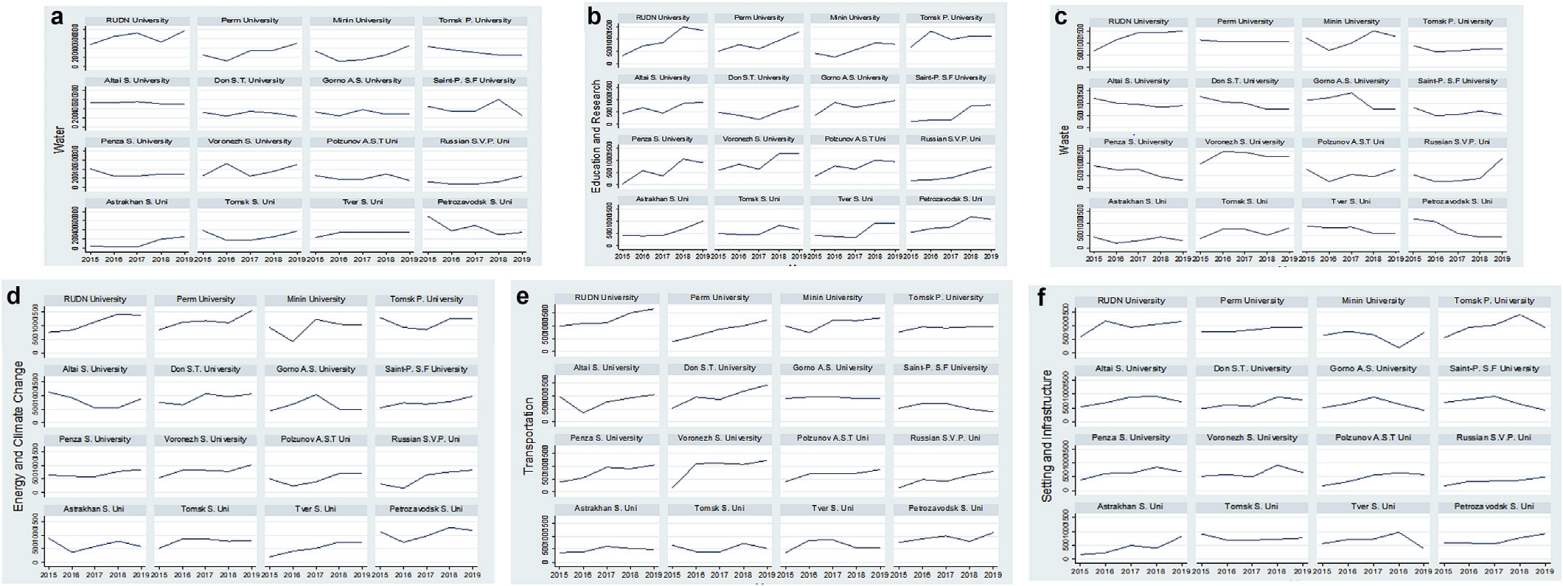
a. Trends of water initiatives in Russian universities from 2015 to 2019. b. Trends of Education and Research initiatives in Russian universities from 2015 to 2019. c. Trends of Waste management initiatives in Russian universities from 2015 to 2019. d. Trends of Energy and Climate change initiatives in Russian universities from 2015 to 2019. e. Trends of Transport initiatives in Russian universities from 2015 to 2019. f.Trends of Settings and Infrastructure initiatives in Russian universities from 2015 to 2019.

Figure 3a–f compares the trends of energy and climate, setting and Infrastructure, education and research transportation, waste, and water by years. The result shows different scenarios in trends over time for the different universities. Whereas some institutions recorded an increase in efforts towards sustainable water management (e.g. RUDN University), others declined (e.g. Petrozavodsk State University) (Figure 3a). Furthermore, the institutions under study generally recorded an improvement in environmental quality proxied as energy and climate change (Figure 3d). However, there were instances where environmental quality declined before picking up. For transportation (Figure 3e), a general increasing trend is observed for all the institutions with only Saint-Petersburg State Forest university observing a generally decreasing trend. Figure 3c further shows that the trend analysis for waste management strongly varies from one institution to the other. For instance, while Minin University and Tomsk State University observed a U-shaped trend, Voronezh State University and Gorno Agrarian State University experienced a zig-zag shaped trend. Finally, the trend analysis for education and research and setting and infrastructure (Figure 3b and Figure 3f respectively) generally shows an upward and downward trend for almost all the universities considered in the study. The trend analysis gives a pictorial view of the general performance of the various universities for all the variables over time.

### 4.3 Diagnostic test

#### 4.3.1 Hausman test

Hausman test (Table 1) was run to decide between fixed-effects and random-effects regressions. The null hypothesis is that the preferred model is random-effects, and the alternative hypothesis is that the preferred model is fixed-effects [38,40]. Hausman is used to test if there is a correlation between the regressors and the unique errors $\left(u_{i}\right)$, stating the null hypothesis as no correlation. As shown in Table 4, the result of the Hausman test indicates that the chi-square is not significant. Thus, we fail to reject the null hypothesis, and conclude that the preferred model is random-effects.

**Table 4:** Hausman test.

Variable	Coefficient		
	(*b*) (Fixed)	*B* (Random)	(*n-B*) Difference
Setting and Infrastructure	-0.1422635	-0.0090538	-0.13321
Transportation	0.2110908	0.1815054	0.029585
Education and Research	0.3476683	0.3223417	0.025327
Waste management	0.2817909	0.238307	0.043484
Water management	0.0113812	0.0983183	-0.08694
*b = consistent under Ho and Ha; obtained from xtreg*			
*B = inconsistent under Ha, efficient under Ho; obtained from xtreg*			
*Test: Ho: difference in coefficients not systematic*			
*chi2(5) = (b-B)'[(V_b-V_B)ˆ(-1)](b-B)*			
*Prob > chi =0.456*			

#### 4.3.2 Test for random effects: Breusch-Pagan LM test

The null hypothesis of the Breusch-Pagan LM test is that variances across entities (universities) are zero. This means that there are no significant differences across units/universities (no panel effect exists). The chi-squared is significant at 1% (Table 5). Hence, the null hypothesis is rejected. Thus, panel effect exists in the data since there are significant differences across countries. It is concluded that random-effects regression is appropriate.

**Table 5:** **Test for random effect with Breusch-Pagan LM test.** Energy and Climate [University, t] = Xb + u[University] + e[University,t].

	Var	sd = sqrt (Var)
Energy and climate	85229.51	291.9409
E	31908.66	178.63
U	14236.77	119.3179
Test:		
Var(u) = 0		
chibar2 (01) = 13.73		
Prob > chibar2 = 0.0001		

Given that both the Hausman test and the Breusch-Pagan test revealed the appropriateness of the random-effects model, we chose the random effects model ahead of the fixed-effects model. However, for reasons of comparison and clarity, the study presented both results.

### 4.4 Linear regression results

Following [44], the random-effects model, and fixed-effects model, were used for the parameter estimation. The results are presented in Table 6.^1^ The F-test and Chi-square are significant at 1%, indicating that the models fit well for the explanation of the data. The adjusted R-squared shows that the explanatory variables used in the fixed-effects, and random-effects models respectively accounted for 38.7 %, and 42.6% respectively of variation in the dependent variable (energy and climate change).

**Table 6:** Estimation results from OLS, fixed-effects, and random-effects models.

Variables	Fixed-effects		Random-effects	
	Coefficient	Standard error	Coefficient	Standard error
Setting and Infrastructure	-0.142	0.133	-0.009	0.125
Transportation	0.211∗	0.113	0.181∗	0.107
Education and Research	0.347∗∗∗	0.092	0.322∗∗∗	0.089
Waste management	0.281∗∗	0.106	0.238∗∗	0.093
Water management	0.011	0.248	0.098	0.215
Constant	257.6948∗∗	127.746	216.731∗∗	105.122
*Observations*	80		80	
*Group Variable*	University		University	
*Number of groups*	16		16	
*Average Observations per group*	5		5	
*F-statistic*	8.06			
*Prob > F*	0.000			
*Wald chi-square*			51.14	
*Prob > chi-square*			0.000	
*Adjusted R-square*				
*Adjusted R-square: within*	0.406		0.392	
*Adjusted R-square: between*	0.373		0.457	
*Adjusted R-square: overall*	0.387		0.426	
*corr(u_i, Xb)*	0.092		0 (assumed)	
*Sigma U*	173.8504		119.317	
Sigma_e	178.630		178.630	
Rho	0.486		0.309	

Note: ∗,∗∗, and ∗∗∗ denote significance at 10%, 5%, and 1% respectively.

The coefficient of transportation is significant in both the fixed-effects and random-effects models. Though the directions of the signs and directions of the coefficients are the same, the magnitudes differ. Specifically, a unit increase in campus-based environmental friendly transport systems leads to an improvement in campus-based environmental quality by 0.21 units (at the 10% level of significance; fixed-effect model). Similarly, a unit increase in campus-based environmental-friendly transport systems will result in a 0.18 unit increase in campus-based environmental quality. This result shows that environmental-friendly transport systems such as the use of bicycle, skating boards, manual and electric scooters to commute within campuses plays a critical role in the reduction of campus-based emission levels that would have otherwise been generated from conventional transport systems.

This finding has important policy implication for Russian universities, the national economy and global economy as a whole. Thus the recent increase in cross-border education especially among HEIs provides a perfect opportunity for the globalization of campus-based sustainability. Foreign students have the opportunity to learn and adopt sustainability initiatives which can be introduced in their home countries upon completion. The idea of Campus-based environmental quality strategies or sustainability is instigated by the realization of direct or indirect adverse effects of HEIs activities on the environment [45]. Successful implementation of campus-based sustainability strategies is solely dependent on the level of commitment and involvement of all stakeholders; which include management, lectures, students, and staff [46, 47, 48]. HEIs due to the nature of activities they undertake, contribute massively to environmental degradation through the emission of CO_2_ gases. This calls for policy interventions that ensure that HEIs are able to minimize the effect of their actions on the environment. A study by [49], identified three campus-based transportation categories: these are i) student transport; ii) transportation by university staff (lecturers,management, and supporting staff); iii) transportation by visitors. These transport categories contribute to the increasing rate of carbon footprint on campuses and therefore result in a reduction in environmental quality. They further observed that whereas emission from student transport was 81 MTCO_2_/year, that from staff was 8.2 MTCO_2_/year and transportation by visitors accounted for 0.86MTCO_2_/year. The forgoing underscores the importance of our finding on transportation. It is important to note that one key indicator for the measurement of sustainable transportation efforts among green/sustainable universities under the greenMetric ranking is “Zero-Emission Vehicles (ZED) policy” on campuses, hence the finding confirms the expectation of the study.

Just as expected, education and research such as the number of sustainability-related programmes run by a university, as well as the number of sustainability-related conferences, lectures, workshops, and sustainability-related scientific publications by a university, significantly influence their improvement in campus-based environmental quality for all three models. Thus, education and research is strongly significant in the fixed-effects, and random-effects model at the 1% significance level. A unit increase in education and research results in a 0.35, and 0.32 unit improvement in environmental quality in the, fixed-effects, and random-effects models respectively. Given that universities are considered as the ultimate agents of change, the organization of sustainability-related programmes provides an excellent platform for all actors in the education system to share ideas about environmental issues and hence develop solutions to solve them. Also, these programmes as mentioned earlier exposes university players to the state of the environment and helps to develop a sense of environmental consciousness among them thereby taking steps on an individual level to help achieve environmental sustainability. Geng et al. [18] argues that with the advent of rapid industrialization and the increasing rate of environmental crisis, governments and industries are demanding that graduates acquire knowledge on broader issues particularly on environmental and sustainability issues. This they observed can be achieved via green education efforts on campuses.

Table 6 further shows that the coefficient of campus-based waste management is significant at 5% and positive for all the three models. The fixed-effects and random-effects models revealed that improving waste management by 1 unit will result in an improvement in environmental quality by 0.28 and 0.24 units respectively. This is not surprising given that some critical indicators for the measurement of waste management efforts on campuses include university recycling waste programmes, organic, inorganic, and toxic waste treatment, as well as programmes that reduce the use of paper and plastics, glass, PET bottles, Styrofoam, food waste, leaves among others on campuses. According to [50], the Asian Institute of Technology (AIT) generates about 1.3 tonnes of waste/day which corresponds to a per capita waste generation of 0.5kg. By extension, this finding presents a clear picture of the amount of waste that is likely to be generated by Russian universities (that is if we assume 1.3 tonnes as the default waste generated by a single university). Ridhosari and Rahman (2019) found that campus-based waste generation in the Universitas Pertamina alone contributes about 14.08MTCO_2_/year to atmospheric CO_2._ They further noted that the total CO_2_ emission from electricity, transport and waste by the Universitas Pertamina amounted to 1351.98 MTCO_2_/year. In effect, the positive nexus between waste management and environmental quality found in this study indicate that the more the waste management initiatives implemented and executed by HEIs, the higher the improvement in the environmental quality of campuses. This means that while HEIs implement strategies that reduce waste production or effectively manage waste, total CO_2_ emission is likely to reduce and hence the resultant improvement in campus-based environmental quality.

### 4.5 Strengths and weaknesses of management practices

**Figure 4:**
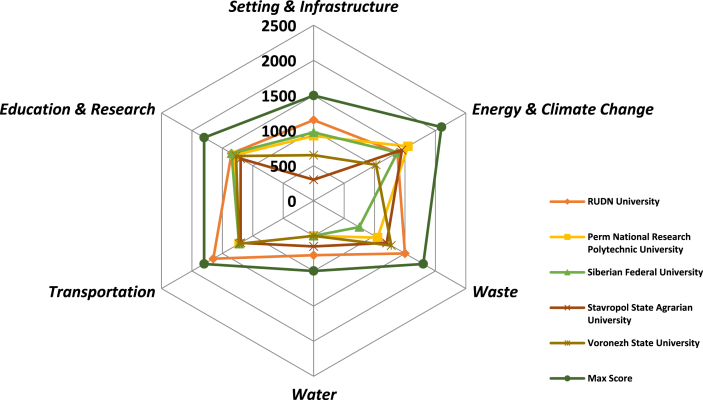
A comparison of the best practices of Russian top 5 sustainable universities.

Analyzing the strengths and weaknesses of the top five participating universities, the accumulated points for each category was compared to the overall total point that could be attained (best practices) under each category (Figure 4). The result shows that whereas the RUDN University applied its best practices in the areas of waste management, emission reduction from campus-based transport systems, and setting and infrastructure, it had noticeable weaknesses in sustainable water management and sustainability in education and research. Perm National Research Polytechnique University had a major strength in Energy and climate change which implies that they have either made a significant effort to ensure the efficient use of energy resources or they have incorporated renewable energy sources into their campus energy generation capacity. The closer a score is to the overall score represented by the outer circle (Best practices) implies the strengths of a given university and the vice versa.

### 4.6 Student perception and awareness of campus stainability

**Figure 5:**
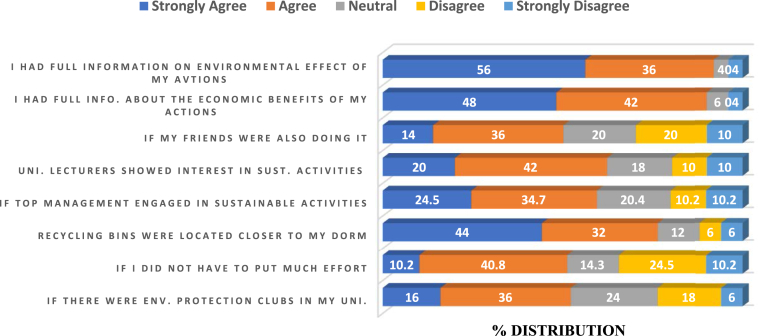
Perception of Students' participation in campus base pro-environmental activities.

Given that HEIs are committed to providing students with specific campus-based opportunities to participate and contribute to make their campuses more sustainable [51], these section complement the study's effort to fully understand the extent of sustainability efforts by Russian universities. Information regarding some influencing factors of pro-environmental activities among students was solicited using a structured questionnaire (see appendix). This helps to appreciate sustainability efforts from students' perspective, thereby enabling universities to improve on future campus-based sustainability policies. Generally, more than half of the student respondents agreed that students likelihood of participation in environmental protection clubs would influence their participation in pro-environmental activities (Figure 5). This finding corroborates [52] who argue that environmental protection clubs present students with the opportunity of “living learning laboratory” system where they are able to work on “real world” projects. They further argue that such clubs could help transition universities into sustainable societies which promotes social justice and incorporates biodiversity principles into socioeconomic development.

Approximately, 5 out of every 10 students agreed to the statement that pro-environmental activities would likely be practiced if less effort is required to do so. Furthermore, the analysis showed that more than half of the respondents agreed to the statement that they would likely engage in pro-environmental activities if they had full information on the environmental effects of their actions. Responding to the question “would you engage in pro-environmental activities if you knew of the economic cost of your action?”, 48% of the respondents strongly agreed.

**Figure 6:**
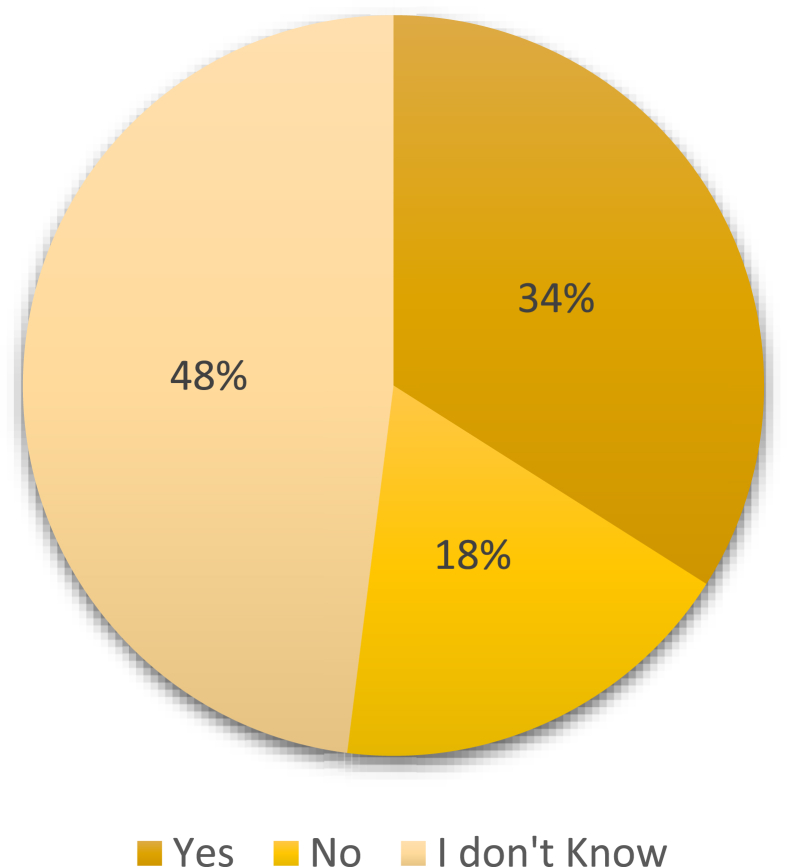
Students' awareness of university environmental policies.

About 59.1% of the respondents agreed to the statement that they will likely engage in pro-environmental behaviours if university management were engaged in sustainability activities. Similarly, 62% of the respondents endorsed the statement that they were more likely to engage in pro-environmental activities if university lectures showed some initiative in this regard. This result concord with [53]. The involvement of university management and staff in campus-based sustainability activities significantly influences others and encourages them to follow suit [54]. Assessing students' awareness of campus-based environmental policies, the majority of the respondents did not know of any such policies in their universities (Figure 6). However, while 36% agreed that such policies existed in their universities, 18% said otherwise. The high percentage of students who did not know of such policies in their universities probably underscores the reason why even though Russian universities undertake campus sustainability, they are still far below the recommended levels for sustainable best practices. This implies that sustainable programs on university campuses are usually one-sided (management-centered).

## 5 Conclusion

Since 2010, several Russian Universities across the various regions in the country have adopted campus sustainability measures to tackle environmental pollution emanating from unsustainable campus activities. The commitment to go green on university campuses is crucial, given the role these institutions play as a driving force of civilization. In light of this, secondary data was used to assess the impact of campus-based management practices on environmental quality, proxied as energy and climate change as well as their strength and weaknesses. Besides, primary data was used to assess some basic pro-environmental awareness initiatives among students.

Findings worth noting are that improvement in education and research, transportation, and waste results in a significant improvement in environmental quality. Also, while the number of sustainable universities in the Russian Federation has increased over the years indicating a growing awareness of universities role in fighting environmental degradation, the general understanding deduced from this paper is that the best sustainable practices of the country's universities are well below that of recommended global standards outlined in the UI GM ranking. The findings further show a general trend where universities tend to priorities more of research and education programmes compared to other indicators under investigation. While this is a good sign, institutions need to strike a balance among the other indicators in other to holistically influence the campus environment sustainably.

Furthermore, the findings reveal that students generally are aware of activities that influence campus-based pro-environmental activities. However, enough efforts have not been made by university management to actively involve students in these initiatives.

To achieve nationwide sustainability on university campuses, the Russian Ministry of Education should mandate all universities to adopt green campus initiatives. This can be done by requiring all Russian universities to undertake campus sustainability reforms that allow university campuses to be used as living laboratories for the teaching and training of students in sustainable related issues. Also, universities must be encouraged to join the GreenMetric world university ranking to create room for regular assessment of green initiatives on university campuses. Also, it is important for university management to actively engage students in every aspect of campus sustainability since they are the main change agents for environmental sustainability for the future.

## 6 Study limitation

Although the study assesses the environmental impact of campus-based management practices by using an econometric model based on panel data, some limitations still exist. For instance, the data used for the study only span 5 years even though the greenMetric ranking dates back to 2010. This is because most Russian university participation in the ranking has been highly inconsistent. Thus, a university may feature in the ranking for some years but not in other years. As a result the study only relied on the existence of consistent data from 2015 to 2019 for the 16 universities considered in the study. Another limitation of the study is the small sample size of the data on the pro-environmental awareness initiatives among students. Since this study is part of an ongoing broad study on campus sustainability, we hope to address these limitations in future studies.

## Declarations

### Author contribution statement

Ernest Baba Ali: Conceived and designed the experiments; Performed the experiments; Analyzed and interpreted the data; Wrote the paper.

Valery P. Anufriev: Conceived and designed the experiments; Wrote the paper.

### Funding statement

This research did not receive any specific grant from funding agencies in the public, commercial, or not-for-profit sectors.

### Competing interest statement

The authors declare no conflict of interest.

### Additional information

No additional information is available for this paper.

## Figures and Tables

**Table:** 

Pro-environmental behaviour: I would engage in pro-environmental behaviour if.	Strongly Agree	Agree	Neutral	Disagree	Strongly Disagree
information about the environmental effects of my actions					
I had full information about the economic benefits of my actions.					
my friends were also doing it.					
university greening lecturers showed interest in sustainable activities.					
top management showed interest in greening (sustainable) activities.					
I would recycle if recycling bins were located closer to my dormitory.					
I didn't have to put much effort.					
there were environmental protection clubs in my university.					

## Appendix

**Survey Questionnaire**

**The following questionnaire is designed to help us understand student awareness and perception of pro-environmental activities on university campuses. While your participation is completely voluntary, your response to the questions will go a long way to help policy directions. It will therefore be much appreciated if you could spare some time to answer the following questions.**

1. Does your university have environmental policies? [1] Yes [2] No [3] I don't know

2. Please read the following statements in the table below and choose form the option on the righthand side of the table. This is to help us understand the perception of students on the pro-environmental behaviour.

## Footnotes

1 Based on the results of the Hausman and Breusch-Pagan LM tests, the study focus estimation results on the random effects model.
